# 

**Published:** 1877-07

**Authors:** 


### EDITORIAL NOTE.

It is scarcely necessary to offer an apology to our many readers, for the exclusion of much from our columns that we trust may be presented hereafter. The full report of the transactions of the AMERICAN MEDICAL ASSOCIATION which we publish in this number, has necessitated the surrender of all available space, even including the additional pages which enlarge this number of the JOURNAL AND EXAMINER.

Since the date of adjournment of the Association left but a brief time for the discharge of the duty which we felt resting upon us, in view of the fact that the Association had met in Chicago, our work has been necessarily hurried. If any errors have been committed, either in names or in expressions, we trust that the haste requisite for the preparation of such an extended report, may be considered in extenuation.

While congratulating therefore our readers upon the fullness with which we have produced an abstract of the proceedings of the Association—proceedings which certainly contain much that is of value and interest—we take pleasure in expressing our thanks to the several gentlemen who have made this effort possible for us, by their kindly assistance. Their names will be found in connection with the report of the section to which each was assigned.

---

### ANNOUNCEMENTS FOR THE MONTH.

SOCIETIES.—Mondays, July 2 and 16; Chicago Medical Society, regular meeting.

Mondays, July 9 and 23; Chicago Society of Physicians and Surgeons.

Friday, July 13; State Microscopical Society of Illinois. Regular meeting at the Academy of Sciences.

CLINICS.—Saturdays at Rush College, 2 P. M. Surgical, Prof. Gunn.

